# MambaVSS-YOLOv11n: State Space Model-Enhanced Multi-Defect Detection in Photovoltaic Module Electroluminescence Images

**DOI:** 10.3390/s26041373

**Published:** 2026-02-21

**Authors:** Kun Wang, Yixin Tang, Xu Wang, Nan Yang, Ziqi Han, Fuzhong Li, Guozhu Song

**Affiliations:** Software College, Shanxi Agricultural University, Jinzhong 030810, China; tangyixin@sxau.edu.cn (Y.T.); wangxu3@sxau.edu.cn (X.W.); nanyang@sxau.edu.cn (N.Y.); ziqihan@sxau.edu.cn (Z.H.); lifuzhong@sxau.edu.cn (F.L.); songgz@sxau.edu.cn (G.S.)

**Keywords:** MambaVSS-YOLOv11n, PV modules, EL images, defect detection

## Abstract

Given the rising global demand for environmentally sustainable energy sources, solar photovoltaic (PV) power generation has emerged as a pivotal component of the energy transition. In PV systems, power conversion efficiency is degraded and operational lifespan reduced due to the presence of defective modules. Consequently, achieving accurate and efficient defect detection during PV module manufacturing is critical to ensuring product quality and reliability. To address this challenge, we propose MambaVSS-YOLOv11n, an electroluminescence (EL) image-based multi-defect detection method for PV modules. Our study utilizes a dataset containing six types of defects—Broken Gate, Cold Solder Joint, Black Spot, Scratch, Microcrack, and Suction Mark—to construct 692 labeled EL images of defective PV modules. The model integrates the Vision State Space (VSS) module from Mamba and optimizes the C3k2 Bottleneck structure to enhance fine-grained feature extraction, while employing Space-to-Depth Convolutional (SPD-Conv) Layer for downsampling to improve computational efficiency. Additionally, to address YOLOv11n’s limited generalization capability for small objects and complex backgrounds, we adopt the Inner Mask Distance Penalized Intersection over the Union (Inner-MDPIoU) loss function, which enhances detection accuracy and mitigates the impact of low-quality samples. Experimental results demonstrate that compared to YOLOv11n, MambaVSS-YOLOv11n reduces the number of parameters by 18.1%, while improving mAP@0.5 to 0.869 and mAP@0.5:0.95 to 0.637. This achieves model lightweighting while enhancing detection performance. These findings indicate that the model is well-suited for real-time defect detection in PV module production lines, providing PV manufacturers with a lightweight yet accurate and reliable solution for PV module defect inspection.

## 1. Introduction

Due to the growing demand for energy and increasing concern for the environment, solar energy has emerged as a critical solution for replacing fossil fuels and reducing carbon emissions, playing a pivotal role in global energy transition and sustainable development [1]. Photovoltaic (PV) power generation, a fundamental approach to harnessing solar energy, involves the direct conversion of solar radiation into electricity through the photovoltaic effect [2]. This technology offers significant advantages including cleanliness, renewability, and widespread availability, promoting the transition of global energy systems. With ongoing technological progress and declining costs, PV power has achieved large-scale global deployment [3]. According to the International Energy Agency (IEA), global PV installations reached approximately 350 GW in 2023, surpassing 1 TW in cumulative capacity, with projections exceeding 3 TW by 2030 [4]. Such rapid growth underscores PV technology’s central role in modern energy infrastructure.

Serving as the core component of PV systems, solar cells directly determine system efficiency and economic viability [5]. Current mainstream technologies include crystalline silicon (monocrystalline and polycrystalline) and thin-film cells, with monocrystalline Passivated Emitter Rear Cell (PERC) cells dominating over 60% of the market at an average conversion efficiency of 23% [6]. However, variations in manufacturing processes often lead to the formation of microscopic defects, material imperfections, or mechanical stresses [7]. Studies indicate that even micron-scale defects can reduce cell efficiency by 0.5–2%, leading to substantial power losses at the module level.

Ensuring component reliability through defect detection remains a significant challenge in quality control for the PV industry [8]. Manual inspections suffer from inefficiency, high costs, and inconsistency, while traditional algorithms struggle to generalize across diverse defect morphologies. Electroluminescence (EL) imaging has become the industry standard for non-destructive testing, capturing defect-induced variations in radiative recombination as characteristic dark spots or abnormal patterns in EL images [9]. This technique provides spatial defect visualization with sensitivity down to 10 μm.

However, emerging trends toward larger wafer sizes and thinner cells impose stricter demands on EL detection [10]. Key challenges include complex defect morphologies (e.g., Broken Gates, poor soldering, black corners, scratches, microcracks) requiring multi-scale feature extraction; low-contrast small targets obscured by background noise; and stringent real-time requirements for production lines exceeding 2000 cells/h. Traditional machine learning methods (e.g., SVM, random forests) struggle with these complexities, and while deep learning has become increasingly prominent, it encounters bottlenecks such as data scarcity, computational overhead, and limited precision [11]. Conventional downsampling strategies (e.g., max pooling, strided convolution) cause feature loss, impairing micro-defect recognition, while complex models suffer from low inference efficiency. High EL data acquisition costs exacerbate overfitting risks, and traditional data augmentation methods prove inadequate for industrial needs [12]. From an industry perspective, manual inspections involve high costs and risks of undetected defects, which can shorten component lifespan and elevate maintenance expenses. These challenges require the development of optimized model architectures and training strategies to improve accuracy, efficiency, and generalization for intelligent quality control. Despite EL imaging’s effectiveness in identifying microcracks and structural defects, the application of advanced, state-of-the-art object detectors (such as the latest YOLO series, DETR, or other modern architectures) to PV EL image defect detection remains limited. This highlights an urgent need for novel approaches and adapted frameworks in this field.

This study proposes the MambaVSS-YOLOv11n model for multi-defect detection in PV EL images, combining the temporal modeling efficiency of MambaVSS with the object detection advancements of YOLOv11n to achieve low-cost, high-precision detection. The primary contributions are:

(1) A novel SPD-Conv-based downsampling method that preserves local spatial information through spatial-to-depth transformation while enhancing multi-scale feature extraction, particularly for subtle defects like Broken Gates. By optimizing computational complexity and feature utilization, SPD-Conv improves detection performance without compromising inference speed, meeting industrial EL inspection requirements.
(2) The C3k2_VSS module, integrating CNN and SSM architectures with a Variable Spatial Scaling (VSS) mechanism, enhances adaptability to multi-scale defects and computational efficiency. This module synergizes local and global features, maintaining sensitivity to details while capturing broader contextual information, thereby improving the detection of large-area soldering defects and microcracks. The dynamic spatial scaling mechanism facilitates feature enhancement and fusion across network layers, outperforming fixed-receptive-field convolutions for EL images with significant scale variations. Additionally, C3k2_VSS reduces redundant computations, balancing high accuracy with efficient inference for industrial applications.
(3) The Inner_MDPIoU loss function accelerates model convergence and enhances generalization capabilities, significantly boosting detection efficiency.

## 2. Related Work

Defect detection in PV modules is a core process for ensuring module reliability and power generation efficiency. Its technological evolution has experienced a paradigm shift from manual inspection to intelligent automated detection. Early detection primarily relied on manual visual inspection, where operators identified defects such as broken grids, poor soldering, and microcracks by analyzing EL or visible-light images [13,14]. However, manual inspection suffers from low efficiency, subjectivity, and high miss rates, making it unsuitable for modern industrial-scale production lines [15]. Rule-based image processing techniques (e.g., edge detection, threshold segmentation, morphological operations) improved automation but remained sensitive to image quality and lacked robustness in low-contrast or complex background scenarios [16,17]. Traditional machine learning methods (e.g., SVM, KNN) achieved classification through manual feature extraction, but their high feature engineering complexity and limited generalization hindered practical applications [18,19]. Multimodal detection techniques (e.g., infrared thermography, ultrasound) can identify deep-layer defects but face challenges in industrial adoption due to high equipment costs and operational complexity [20,21]. The core limitation of these traditional approaches lies in their inability to autonomously extract deep semantic features from images, failing to address complex and variable defect patterns.

The YOLO (You Only Look Once) series, as a representative single-stage object detection framework, has been widely adopted in industrial quality inspection owing to its end-to-end architecture and efficient inference speed [22]. From YOLOv1 to YOLOv10, through successive iterations, significant advancements have been achieved in multi-scale detection capability and lightweight design through structures like CSPNet, PAN feature pyramids, and attention mechanisms [23]. Current research on PV module defect detection predominantly focuses on earlier YOLO versions (e.g., YOLOv5, YOLOv8). For instance, YOLOv5 improved defect detection accuracy by integrating a weighted bidirectional feature pyramid [24], while YOLOv8 achieved high robustness in complex scenes using a background noise suppression module [25]. The recently proposed YOLO11 further balances global feature modeling and local detail capture by combining CNN and Transformer architectures, achieving state-of-the-art (SOTA) performance in general object detection tasks. Preliminary experiments indicate that YOLO11 outperforms previous models in detection accuracy (mAP@0.5) on PV module EL images. However, it still exhibits high miss rates for microscopic defects (e.g., microcracks, Suction Marks) and boundary localization errors [26]. Yu et al. proposed a multimodal fault detection model, which effectively improves the overall recognition accuracy of defects in complex backgrounds by fusing features from visible light and infrared images [27]. Jabrane et al.’s work focuses on real-time performance; they employ a lightweight Vision Transformer (ViT) to directly process thermal images on drone edge devices, successfully achieving rapid hotspot localization [28]. Cao et al. designed a lightweight Transformer network (LWMF-DETR), which innovatively introduces multi-scale wavelet feature fusion to enhance feature extraction capability for tiny hot-spot faults, thereby improving detection sensitivity [29]. Moreover, PV module EL images often suffer from low contrast, noise interference, and diverse defect morphologies (e.g., irregular microcracks, extended broken grids). YOLO11’s fixed receptive field design struggles to capture long-range dependencies, leading to insufficient fusion of local features and global contextual information [30]. Traditional convolutional operations’ sensitivity to scale variations also limits the model’s ability to detect defects of varying sizes [31].

To overcome the performance limitations of YOLO-series models, researchers are exploring novel architectures and hybrid paradigms. The Mamba model, with its Selective State Space mechanism and hardware-aware optimization, demonstrates significant advantages in long-sequence modeling and computational efficiency [32,33,34]. Its core innovation involves dynamically adjusting information attention weights, effectively filtering background noise while enhancing critical feature representation. This approach has proven effective in tasks such as medical image segmentation and drone-based object detection [35,36,37,38]. For example, MambaCrackNet improved crack segmentation accuracy using residual visual Mamba modules [37], and YOLO-Mamba optimized feature extraction for nighttime infrared detection by integrating attention mechanisms [38]. Zhao et al. employed the Visual State Space Model (VSSM) as the backbone network in the YOLO detection framework, effectively aggregating global multi-scale contextual information. This approach addresses the limited receptive field of traditional CNNs, enhances feature representation for each pixel, and improves the efficiency of small object detection [39].

## 3. Experimental Dataset Construction

This study follows a clear technical workflow: First, EL image data of monocrystalline PV modules is acquired. Then, the processed data is input into a convolutional neural network for model training and optimization. The optimized model undergoes performance evaluation on test data, and finally, predictions for target objectives are made based on the evaluation results.

### 3.1. Data Preparation

**Figure 1:**
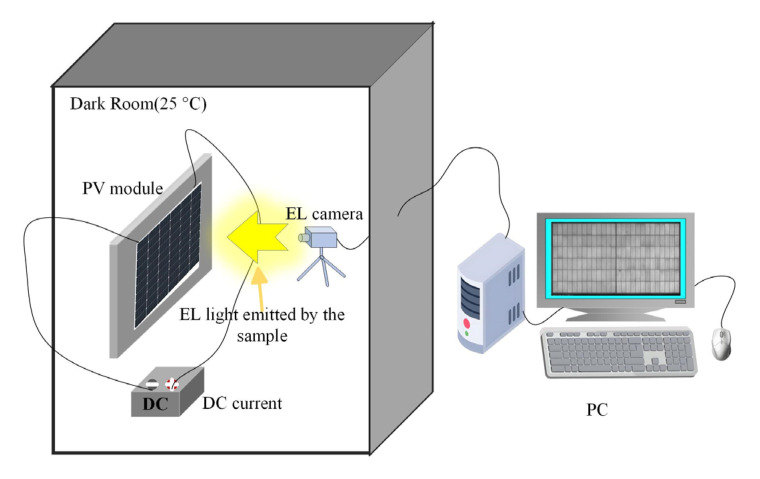
EL Image Acquisition Device Schematic.

The data used for training and evaluating the PV panel defect detection model is typically collected via EL imaging technology. During the data collection process, different types of defects—including “Broken Gate, Cold Solder Joint, Black Spot, Scratch, Microcrack, and Suction Mark”—are first categorized to ensure data diversity, covering defects of varying degrees and morphologies. Subsequently, high-resolution defect images are acquired through EL imaging to accurately reflect the visual characteristics of the defects. The EL images were captured using an infrared spectrum camera under the standard test current Isc with an exposure time of 50,000 ms, yielding an image resolution of 6180 × 3838 pixels. The equipment for EL image data acquisition is illustrated in Figure 1.

**Figure 2:**
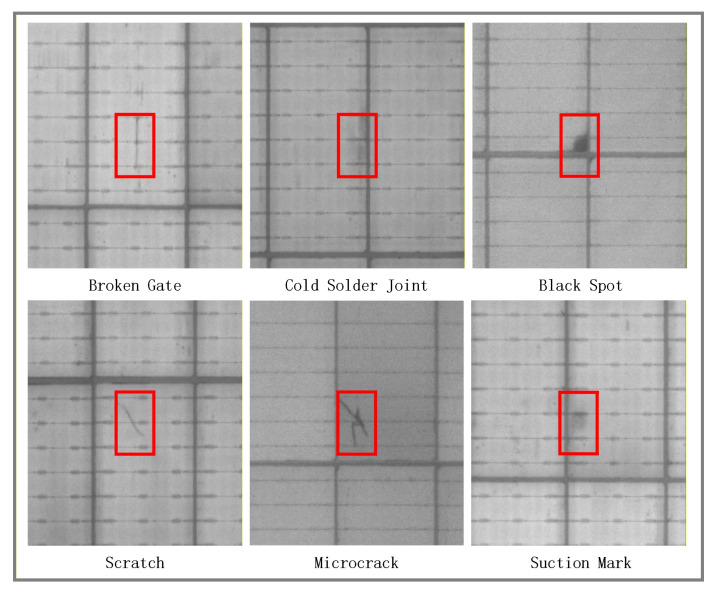
Photovoltaic Module Defect Schematic Diagram.

The typical defect types in PV modules include “Broken Gate, Cold Solder Joint, Black Spot, Scratch, Microcrack, and Suction Mark”, with their characteristic morphologies illustrated in Figure 2. These defects exhibit significant differences in morphology, brightness distribution, and edge characteristics within EL images. The defect types and their descriptions are detailed in Table 1.

### 3.2. Micro-Defect Annotation in Captured Images

In this experiment, we utilized the LabelImg annotation tool to complete the dataset labeling task. The process primarily involved four steps: data preparation, labeling standard formulation, image annotation, and result validation. First, representative defect samples were selected from the experimentally collected EL images. A detailed labeling standard was established based on defect characteristics, requiring bounding boxes to tightly enclose defect regions to ensure complete capture of defect information while minimizing background noise. Second, each image was manually annotated using LabelImg. The annotation work was performed by five trained annotators according to the defect characteristic criteria specified in Table 1. Ultimately, the training dataset comprises a total of 1635 defect samples, with the following specific distribution across categories: 253 instances of Broken Gate, 222 instances of Cold Solder Joint, 198 instances of Black Spot, 816 instances of Scratch, 13 instances of Microcrack, and 133 instances of Suction Mark. To mitigate the adverse effects of imbalanced data distribution on model training, oversampling is applied to categories with fewer samples in each epoch. This ensures that during batch training, the probability of each class being seen by the model becomes more balanced.

**Figure 3:**
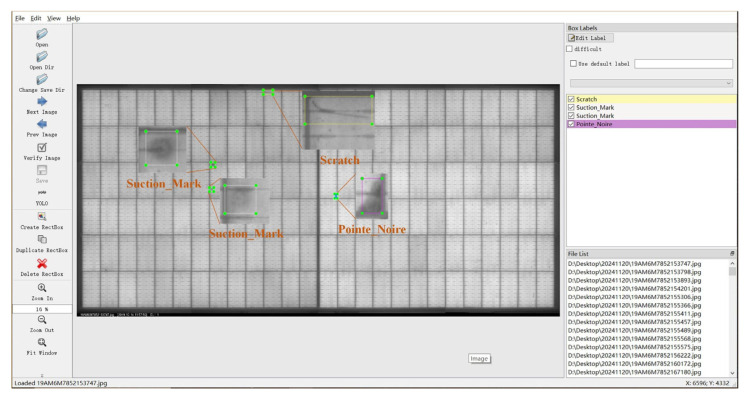
Defect Annotation in Image Data Samples.

During annotation, auxiliary features such as zooming and panning were employed to precisely draw bounding boxes around each defect. Defects were assigned numerical labels according to a predefined classification system: 1 for Broken Gate, 2 for Cold Solder Joint, 3 for Black Spot, 4 for scratch, 5 for Microcrack, and 6 for Suction Mark. After annotation, the information of all bounding boxes—including normalized coordinates (x_center, y_center, width, height) and class labels—was recorded in separate TXT files. This ensured data integrity and consistency, providing high-quality and reliable samples for subsequent model training. A schematic of the sample annotation process is shown in Figure 3.

### 3.3. Photovoltaic Module Defect Image Dataset Construction

This study employs offline data augmentation techniques on the collected data samples. Specifically, we adopt augmentation strategies such as flipping, brightness adjustment, contrast adjustment, blurring, and noise addition. By horizontally or vertically flipping images, we simulate appearance variations in defects under different imaging angles, enhancing the model’s adaptability to directional changes. Brightness adjustment improves the model’s robustness to defect features under varying illumination conditions. Contrast adjustment enhances the model’s ability to discern edge characteristics of defects, addressing impacts caused by imaging equipment or environmental differences. Gaussian blur or motion blur is introduced to partially obscure defect regions, strengthening the model’s stability when processing low-quality or slightly out-of-focus images. Gaussian noise or salt-and-pepper noise is added to improve the model’s resilience against sensor noise and image transmission errors. These data augmentation strategies effectively expand the diversity of data distributions, enabling the model to learn defect features more comprehensively and thereby improving detection accuracy and generalization capability. Additionally, to address significant class imbalance among different defect types, an oversampling strategy is applied to defect categories with fewer samples, increasing their proportion in the training set to mitigate performance degradation caused by class imbalance during training.

The annotated dataset of 692 defect samples is divided into a training set and a validation set at an 8:2 ratio. Specifically, 80% of the data is used for training, allowing the model to thoroughly learn features of various defects and enhance its capability to detect subtle defects in complex scenarios. The remaining 20% serves as the validation set, providing independent evaluation data for model assessment.

## 4. Model Composition and Improvement Description

This section provides a detailed overview of the experimental design and implementation details of the study. Section 4.1 details the composition of the proposed model MambaVSS-YOLOv11n, including the YOLOv11n base architecture, the C3k2_VSS module, the SPD-Conv module, and the Inner-MDPIoU loss function. Section 4.2 provides a comprehensive analysis of the structural and algorithmic improvements integrated into the MambaVSS-YOLOv11n framework. Finally, Section 4.3 details the configuration of the model training platform and the settings of network hyperparameters.

### 4.1. Introduction to Existing Models

#### 4.1.1. YOLOv11n Model

**Figure 4:**
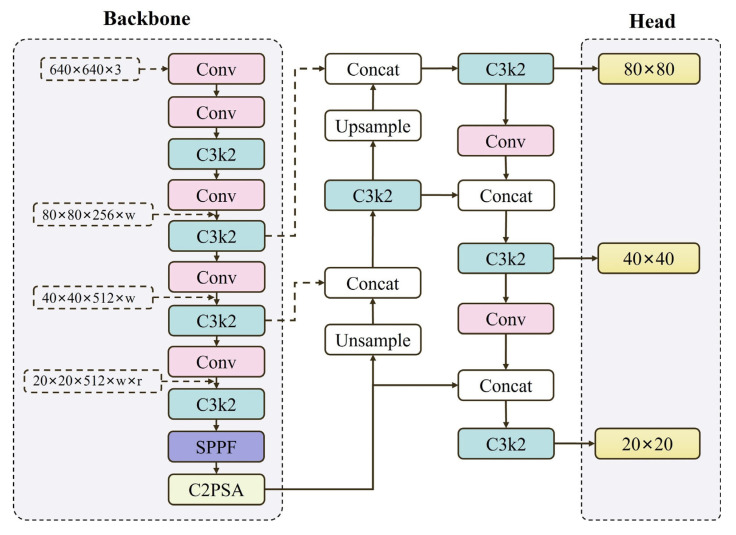
YOLOv11n Model Structure Diagram.

The YOLOv11 [40] series provides five model variants of different scales (n, s, m, l, x), with increasing depth, width, and parameter count. Although the l and x variants achieve higher mAP on public datasets, their substantial parameter counts and FLOPs make them difficult to meet the stringent real-time requirements of edge computing devices on PV production lines. In comparison, YOLOv11n has the smallest parameter count (approximately 2.6 M) and the fastest inference speed. Considering the practical application scenario of this study, we selected YOLOv11n as the baseline model. The architecture of YOLOv11n integrates three core components: a backbone network for hierarchical feature extraction, a neck structure for multi-scale feature fusion, and a detection head for final localization and classification tasks. The backbone network employs critical modules including Conv, C3k2, SPPF, and C2PSA, with the latter—introduced after the SPPF module—combining two standard convolutional layers and a multi-head self-attention mechanism to enhance deep semantic feature representation. The neck structure, augmented with the C3k2 module (a derivative of the C2f module), utilizes multi-scale convolutional kernels and channel grouping strategies to split input features into dual branches: one processed via standard convolution and the other refined through stacked C3k modules or Bottleneck structures. These parallel outputs are concatenated and fused via a 1 × 1 convolutional layer, yielding discriminative feature maps. Notably, the C3k module supports configurable kernel sizes (e.g., 3 × 3, 5 × 5) to dynamically expand the model’s receptive field, thereby improving target detection in cluttered environments. A hyperparameter-controlled design allows switching between C3k and default C2f configurations (C3k = True/False), ensuring adaptability across diverse scenarios. In the detection head, depth-wise separable convolutions replace conventional layers in the classification branch, drastically curtailing computational overhead while maintaining accuracy. The complete architecture of YOLOv11n is depicted in Figure 4.

#### 4.1.2. C3k2_VSS Model

**Figure 5:**
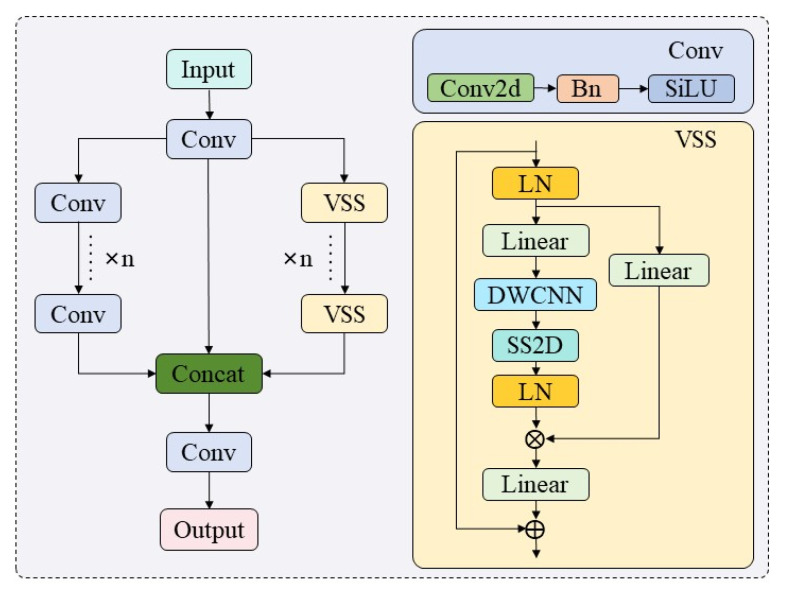
C3k2_VSS Module Structure Diagram.

**Figure 6:**
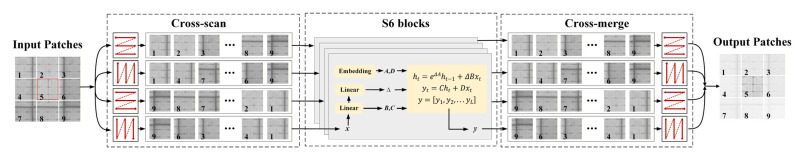
SS2D Working Principle.

The C3k2_VSS module integrates CNN and SSM architectures to simultaneously capture local fine-grained features and long-range dependencies in images, as illustrated in Figure 5. This three-branch module first processes the input feature map through a 1 × 1 convolutional block, splitting it into three identical sub-inputs. Two sub-inputs are routed to parallel branches: a Conv branch and an SSM branch (implemented via VSS [41]), while the third sub-input serves as a residual pathway. In the Conv branch, sequential 1 × 1 CBS and 3 × 3 CBS blocks extract localized spatial patterns. The SSM branch employs a dual-path design: the first path processes feature through Linear and SiLU layers, while the second path applies Linear, Depthwise Convolution(DW-Conv), and activation functions before feeding features into SS2D, SS2D first unfolds the input patches into sequences along four different traversal paths, processes each patch sequence in parallel using separate S6 modules, and subsequently reshapes and merges the resulting sequences to form the output feature map. Its purpose is to adaptively enhance defect-related features and suppress irrelevant background noise, which significantly improves the discernibility of tiny, low-contrast defects such as microcracks and broken fingers in complex EL images. As illustrated in Figure 6, the defect region in the input patches is enclosed by a red box. After feature transformation, all regions other than Region 5 are found to be attenuated. Both SSM sub-branches undergo Layer Normalization (LN), with their outputs merged via element-wise multiplication, followed by Linear transformation and residual addition to generate the VSS output. Finally, the Conv branch features, SSM-processed features, and residual sub-input are concatenated along the channel dimension, followed by 1 × 1 convolutional fusion to enhance cross-feature interaction. This hybrid design enables synergistic learning of detailed texture cues (via CNN) and global contextual relationships (via SSM), making it particularly effective for analyzing EL images of PV modules.

#### 4.1.3. SPD-Conv Model

**Figure 7:**
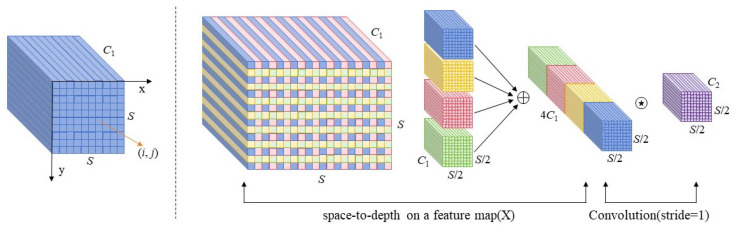
SPD-Conv Module Structure Diagram.

As the network deepens, the model gradually performs downsampling operations on the input image to obtain deep feature maps. In PV module defect detection, since defect targets are small and exhibit low contrast against the background, they are difficult to distinguish. This makes it challenging for the model to separate defect features from the background during feature extraction, limiting the contextual information the model needs to learn and leading to significant false positives and missed detections. The role of the SPD layer is to reduce the spatial dimensions of the input feature map into the channel dimension while ensuring no loss of channel information. To mitigate potential over-sampling in the SPD layer, the Conv layer performs standard convolution operations by processing each pixel or feature map, thereby preserving substantial fine-grained information. The specific process is shown in Figure 7.

The transformation process of SPD-Conv [42] is as follows: First, the SPD layer downsamples the input feature map “X” (size: S × S × C_1_) by a scale factor to obtain sub-feature maps with dimensions (S/scale, S/scale, C_1_). Then, all sub-feature maps are concatenated along the channel dimension to generate an intermediate feature map X′ with dimensions (S/scale, S/scale, 2 × scale × C_1_). Finally, the intermediate feature map X′ is fed into a non-strided convolution layer containing D filters (D < 2 × scale × C_1_), transforming the intermediate feature map into dimensions (S/scale, S/scale, D).

#### 4.1.4. The Loss Function Is Replaced with Inner-MDPIoU

To improve target localization accuracy, YOLO11 adopts CIoU as the bounding box regression loss function. This method optimizes DIoU (Distance Intersection over Union) by introducing an additional aspect ratio penalty term, thereby enhancing constraints on matching target dimensions and shapes to boost overall detection performance. However, CIoU still exhibits limitations in small target detection tasks. First, this loss function inadequately incorporates the intrinsic scale and geometric characteristics of target boxes, over-relying on the distance between predicted and ground-truth box centers to quantify localization errors. For small targets, even minor center offsets can cause drastic loss fluctuations, destabilizing the regression process. Additionally, since precise delineation of target contours is challenging, their features are prone to degradation in overlapping regions, further compromising bounding box regression accuracy and constraining model performance in small target detection scenarios. To address this, we propose a novel loss function named Inner-MDPIoU, which combines the strengths of Inner-IoU and MDPIoU. This enhancement significantly improves YOLOv11n’s detection accuracy, particularly for complex and hard-to-localize targets.

*MPDIoU* considers overlapping/non-overlapping regions, center distance, and width/height deviations while simplifying computation as shown in Equations (1) and (2):

(1) $$MPDIoU\;=\;IoU\;-\frac{d_{1}^{2}+\;d_{2}^{2}}{\left(h^{2}+\;w^{2}\right)}$$

(2) $$L_{MPDIoU}=1-MPDIoU$$

where *d_1_* and *d_2_* represent the Euclidean distances between the diagonals of predicted and ground-truth bounding boxes, *h* and *w* denote the height and width of the bounding boxes, and *IoU* indicates the Intersection over Union between predicted and ground-truth boxes.

To accelerate MDPIoU’s convergence, we introduce Inner-IoU’s auxiliary bounding box concept defined by:

(3) $$b_{l}^{gt}=\;x_{c}^{gt}-\frac{w^{gt}*\;ratio}{2},\;\;b_{r}^{gt}=\;x_{c}^{gt}+\frac{w^{gt}*\;ratio}{2}$$

(4) $$b_{t}^{gt}=y_{c}^{gt}-\frac{h^{gt}*\;ratio}{2},\;\;b_{b}^{gt}=y_{c}^{gt}+\frac{h^{gt}*\;ratio}{2}$$

(5) $$b_{l}=x_{c}-\frac{w\;*\;ratio}{2},\;\;b_{r}=x_{c}+\frac{w\;*\;ratio}{2}$$

(6) $$b_{t}=y_{c}-\frac{h\;*\;ratio}{2},\;\;b_{b}=y_{c}+\frac{h\;*\;ratio}{2}$$

(7) $$inter=\left(\min\left(b_{r}^{gt},\;b_{r}\right)-\max\left(b_{l}^{gt},\;b_{l}\right)\right)\times \left(\min\left(b_{b}^{gt},\;b_{b}\right)-\max\left(b_{t}^{gt},\;b_{t}\right)\right)$$

(8) $$union=\left(w^{gt}\times h^{gt}\right)\times ratio^{2}+\left(w\times h\right)\times ratio^{2}-inter$$

(9) $$IoU^{inner}=\frac{inter}{union}$$

(10) $$L_{Inner-MPDIoU}=L_{MPDIoU}+IoU-IoU^{inner}$$

where $b_{l}^{gt}$, $b_{r}^{gt}$, $b_{t}^{gt}$, $b_{b}^{gt}$ are left/right/top/bottom coordinates of the ground-truth box; $b_{l}$, $b_{r}$, $b_{t}$, $b_{b}$ are corresponding coordinates of the predicted box; $x_{c}^{gt}$, $y_{c}^{gt}$ and $x_{c}$, $y_{c}$ are center coordinates of ground-truth and predicted boxes; $w^{gt}$, $h^{gt}$ and w, h are their widths and heights; ratio is a scaling factor (typically in [0.5, 1.5]) adjusting the ground-truth box size; inter and union are intersection and union areas; $IoU^{inner}$ is the intersection-over-union ratio for auxiliary boxes; and $L_{Inner-MPDIoU}$ is the proposed loss function.

*Inner-MDPIoU* enhances regression accuracy via multi-scale auxiliary bounding boxes. During computation, samples are first categorized by their *IoU* values (overlap degree). For high-IoU samples (indicating precise localization), smaller auxiliary boxes accelerate regression while preventing over-adjustment. Conversely, low-IoU samples (poor localization) employ larger auxiliary boxes to broaden spatial scope for effective correction. By integrating scale factor ratio to generate dynamically scaled auxiliary boxes, *Inner-MDPIoU* addresses limitations of traditional IoU-based regression methods. This approach accelerates bounding box regression, reduces over-reliance on singular loss terms, and effectively promotes model convergence, ultimately boosting detection performance and generalization capability in PV panel defect inspection scenarios.

### 4.2. Overall Architecture of the Improved MambaVSS-YOLOv11n Model

**Figure 8:**
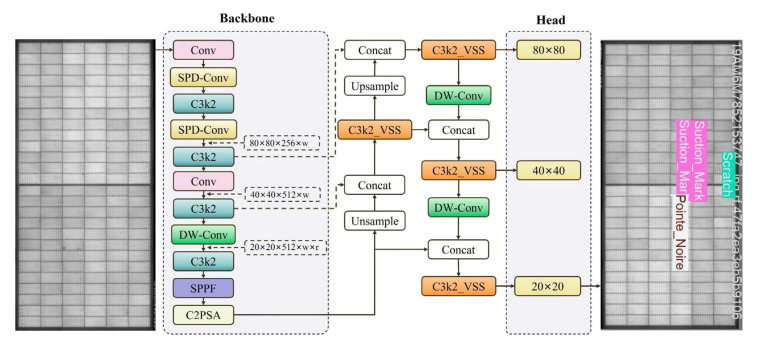
MambaVSS-YOLOv11n Module Structure Diagram.

This study builds upon the YOLOv11n model, tailored for photovoltaic module defect detection. First, SPD-Conv is employed for downsampling, enriching the overall model with enhanced fine-grained information and improving the backbone network’s capability to extract multi-scale features from PV panel images. Subsequently, a C3k2_VSS module is proposed by integrating CNN architecture with SSM (State Space Model) structure. This module processes input features from diverse perspectives, enabling comprehensive extraction of heterogeneous feature representations while minimizing interference from irrelevant information. This design facilitates accurately capturing complex interdependencies between targets, thereby elevating the model’s semantic comprehension of images. Finally, the loss function is replaced with Inner_MDPIoU to accelerate convergence and strengthen generalization capability. Additionally, for the final target determination during the inference stage, this study implements a rigorous post-processing mechanism at the output of the three detection heads with different scales (80 × 80, 40 × 40, 20 × 20). This process consists of three key steps: first, decoding, which maps the raw feature map offsets output by the detection heads back to original image coordinates (cx, cy, w, h) using an Anchor-free formulation; second, confidence filtering, where a threshold (conf_thres = 0.25) is applied to eliminate background or low-confidence candidate boxes; and finally, non-maximum suppression (NMS), which selects the highest-scoring bounding boxes locally through an IoU threshold (iou_thres = 0.7). This mechanism ensures that the model can precisely output the unique locations of defects from multi-scale features, effectively addressing the final decision-making challenge after multi-scale feature fusion. The enhanced architecture is illustrated in Figure 8.

The improved MambaVSS-YOLOv11n model significantly enhances object detection performance by integrating the VSS module from Mamba-UNet, SPD-Conv downsampling technique, and Inner-MDPIoU loss function into the YOLOv11n architecture. Specifically, the VSS module enhances the Bottleneck layers in C3k2, enabling the model to more effectively capture image details and broad semantic contexts. SPD-Conv replaces traditional downsampling methods through multi-scale feature fusion and dilated convolutions, improving model accuracy and robustness. DW-Conv is a depthwise separable convolution that significantly reduces the computational cost and number of parameters in the model. The Inner-MDPIoU loss function optimizes the matching precision between predicted and ground-truth bounding boxes, particularly boosting localization accuracy for small objects and complex targets. These enhancements allow MambaVSS-YOLOv11n to demonstrate superior performance in complex detection tasks, especially in handling small objects, blurred boundaries, and multi-scale images, exhibiting stronger adaptability and accuracy.

### 4.3. Model Training Platform and Hyperparameter Configuration

#### 4.3.1. Training Platform Introduction

This study utilizes GPU acceleration for dataset training, employing an NVIDIA RTX 4090 graphics card with 90 GB of memory. Detailed specifications of other hardware/software components and their respective models are provided in Table 2.

#### 4.3.2. Network Training Hyperparameter Configuration

The hyperparameter configurations for training the proposed network model are summarized in Table 3. The learning rate is dynamically adjusted using a cosine annealing scheduler to balance rapid convergence in early training stages and fine-grained optimization in later phases. The optimizer operates in Auto mode, where the framework automatically selects the optimal parameter update strategy (e.g., AdamW or SGD-Momentum) based on gradient distribution characteristics, thereby enhancing training stability. The training process spans 200 epochs with a batch size of 32, ensuring high GPU memory utilization while improving the statistical reliability of gradient estimation. To prevent overfitting and reduce computational redundancy, an early stopping mechanism (patience = 50) is implemented, which terminates training if validation metrics show no improvement for 50 consecutive epochs. Model initialization employs random weights (pretrained = False) to mitigate negative transfer effects caused by domain discrepancies between pretrained weights and PV EL images.

## 5. Results and Discussion

### 5.1. Evaluation Metrics

To accurately evaluate model performance, this study employs Precision, Recall, and mean Average Precision (mAP, including mAP@0.5 and mAP@0.5:0.95) as the core evaluation metrics. The mAP is derived by averaging the area under the Precision-Recall curve across all categories; mAP@0.5 denotes the mean Average Precision at an Intersection over Union (IoU) threshold of 0.5, while mAP@0.5:0.95 represents the average mAP over IoU thresholds ranging from 0.5 to 0.95 with a step size of 0.05. The computational methods for Precision, Recall, and mAP are defined by Equation (11), Equation (12), and Equation (13), respectively:

(11) $$\mathrm{Precision}\;=\;\frac{TP}{TP+FP}$$

(12) $$\mathrm{Recall}=\frac{TP}{TP+FN}$$

(13) $$\mathrm{mAP}=\frac{1}{C}\sum_{i=1}^{C}AP_{i}$$

where *TP* denotes the number of True Positives, *FP* denotes the number of False Positives, *FN* denotes the number of False Negatives, *C* represents the total number of categories, and $AP_{i}$ denotes the Average Precision value for the i-th class.

### 5.2. Comparative Experiment

To further validate the effectiveness of the proposed improvement method, we selected current mainstream object detection models including YOLOv5n, YOLOv6n, YOLOv8n, YOLOv9t, YOLOv10n, and YOLOv11n as comparison subjects. Each baseline model was trained using its officially recommended hyperparameter configuration and data-augmentation strategy to ensure a fair and near-optimal performance comparison, with experimental results shown in Table 4.

While different versions of the YOLO series models (e.g., YOLOv6, YOLOv8, etc.) theoretically possess their own optimal hyperparameter spaces, this experiment aims to investigate the “out-of-the-box adaptability” and robustness of the models under limited computational resources and standardized industrial deployment scenarios. This setup simulates the practical constraints in real-world industrial applications, where engineers often struggle to perform exhaustive hyperparameter searches for each candidate model. It is noteworthy that the performance of YOLOv6n in Table 4 (mAP@0.5 of 0.566) is lower than that of other comparative models. This phenomenon can be attributed to the high sensitivity of the YOLOv6 series architecture to specific training techniques (e.g., certain reparameterization training recipes, longer warm-up periods, or specific anchor alignment strategies). Under the unified experimental constraints, it failed to achieve optimal convergence, which in turn reveals its generalization limitations when faced with non-customized training strategies. In contrast, the proposed MambaVSS-YOLOv11n, as well as models such as YOLOv5n and YOLOv9t, all achieved superior performance under the same standardized settings, demonstrating stronger robustness of these architectures to hyperparameter variations in the task of PV defect detection.

**Figure 9:**
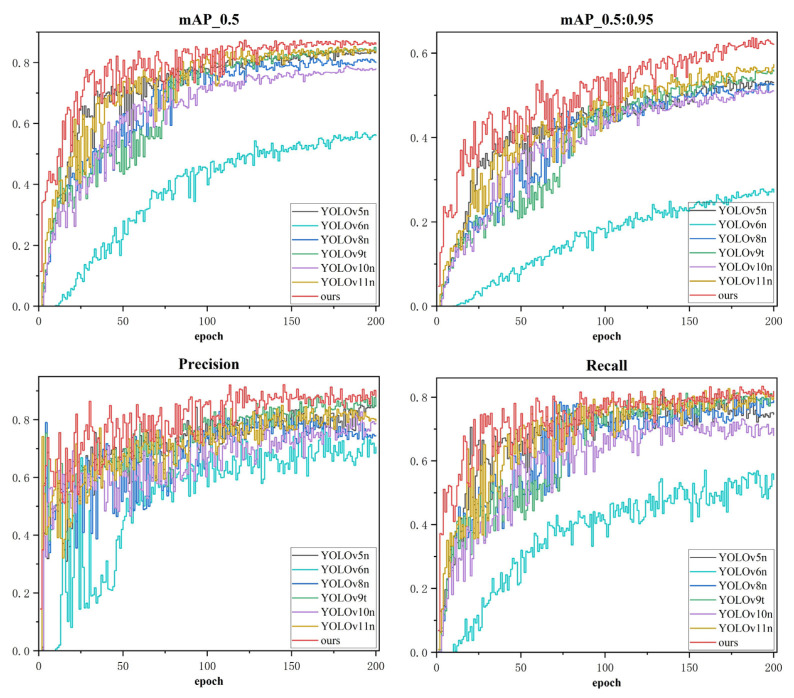
Performance Metrics Evolution of Different Models During Training Phase.

Notably, as one of the latest generation representatives, YOLOv11n demonstrated strong competitiveness, with outstanding mAP0.5 (0.833) performance, particularly its mAP0.5:0.95 (0.572) significantly outperforming all other comparison models except our method. This fully demonstrates that YOLOv11n is an excellent and efficient strong baseline model. Compared to all the above models, our proposed improved model (ours) achieved optimal performance across all metrics: Precision of 0.872, Recall of 0.819, mAP0.5 and mAP0.5:0.95 of 0.861 and 0.623 respectively. Compared to the baseline model YOLOv11n, our model achieved comprehensive performance improvements: Precision increased by 0.078 (7.8%), Recall improved by 0.017 (1.7%), mAP0.5 enhanced by 0.028 (2.8%), and mAP0.5:0.95 significantly increased by 0.051 (5.1 percentage points). Although the computational cost increased from YOLOv11n’s 6.4 G to 8.0 G, it remained lower than YOLOv6n (11.5 G) and YOLOv8n (8.2 G), and was comparable to YOLOv5n (7.8 G) and YOLOv9t (7.6 G), maintaining good computational efficiency. The improvement in mAP0.5:0.95 was particularly significant, showing a 6.3 percentage point increase (0.623 vs. 0.560) compared to the second-best YOLOv9t, further highlighting the effectiveness of our optimization based on YOLOv11n. Therefore, our improved model achieves significant enhancements in detection accuracy and generalization capability while maintaining model lightweightness (Params only 2.12 M, lower than YOLOv11n’s 2.59 M) and relatively high computational efficiency (FLOPs of 8.0 G), demonstrating excellent comprehensive performance and practical application value. Finally, we conducted a quantitative comparison and analysis of the inference speeds of all models on the current experimental platform. All inference tests were carried out on an NVIDIA RTX 4090 GPU with an input resolution of 640 × 640 pixels. The results indicate that the inference efficiency of the model is closely correlated with its computational complexity. Among them, YOLOv11n achieves the highest inference speed of 315 FPS due to its minimal FLOPs. Benefiting from the efficient optimization strategy, although our model has a slight increase in computational cost compared with the baseline, it still maintains a superior inference speed of 288 FPS, it satisfies the real-time demand for PV detection. The training phase metric variations for each model are shown in Figure 9.

From the precision and recall curves, it can be observed that the improved model demonstrates faster convergence speed in the early training stage, with minimal fluctuations and a more stable upward trend. In the middle and later stages of training, both its precision and recall stabilize at high levels (approximately 0.872 and 0.819, respectively), significantly outperforming other baseline models, reflecting stronger discriminative and recall capabilities. In the mAP@0.5 curve, the improved model establishes a performance gap with other models from the early training phase and maintains this lead throughout, ultimately stabilizing around 0.861—a clear improvement even over the relatively strong YOLOv9t model. Particularly under the stricter evaluation metric mAP@0.5:0.95, the advantages of the improved model are even more pronounced, achieving a final value of approximately 0.623, far surpassing models such as YOLOv5n, YOLOv8n, and YOLOv10n. This highlights its enhanced robustness in handling scale variations and complex backgrounds, as well as the high convergence stability exhibited during training. Moreover, all performance curves on the validation set remain at high levels without significant oscillations. Additionally, by employing an early stopping mechanism (patience = 50) and a cosine annealing learning rate scheduling strategy, we effectively suppress random fluctuations caused by overfitting or non-convergence, ensuring that the reported metrics reliably reflect the true performance of the converged model.

**Figure 10:**
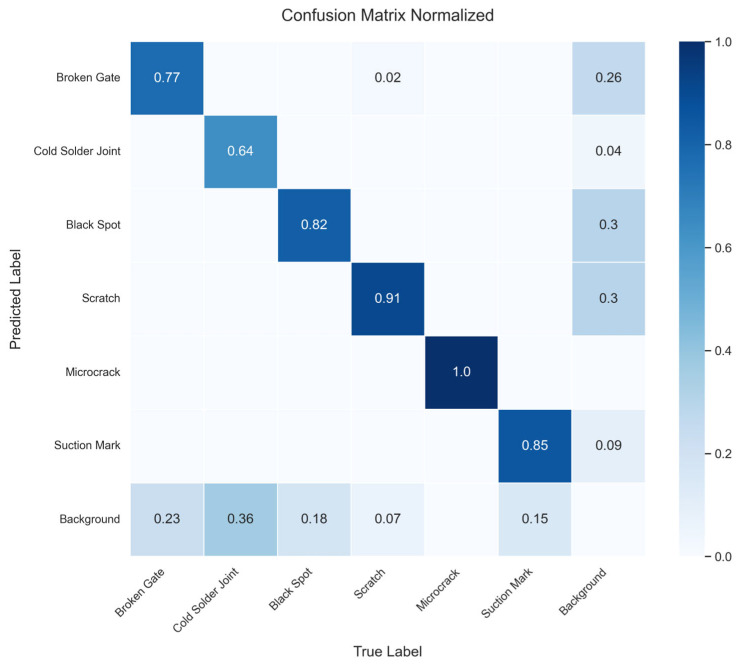
Confusion Matrix Normalized.

Furthermore, to evaluate the specific classification performance and misjudgment of MambaVSS-YOLOv11n on various defect types, we plotted the normalized confusion matrix on the test set, as shown in Figure 10. The diagonal values of the confusion matrix represent the recall rate for each defect category, reflecting the model’s ability to correctly identify that type of defect. As can be observed from the figure, the model demonstrates exceptionally strong recognition performance for the Microcrack and Scratch categories, achieving recall rates of 1.00 and 0.91, respectively. This notable outcome strongly validates the effectiveness of the introduced C3k2_VSS module in handling features with long-range dependencies (such as continuous crack lines and scratch paths), where the SSM mechanism successfully captures the global semantic information of these elongated defects. Meanwhile, the recognition rates for Suction Mark and Black Spot also remain at relatively high levels, at 0.85 and 0.82, respectively. However, the confusion matrix also reveals detection challenges for certain defects. The recall rates for Cold Solder Joint and Broken Gate are relatively lower, at 0.64 and 0.77, respectively. Analysis of the off-diagonal elements indicates that these two defect types are mainly misclassified as Background, with false detection rates of 0.36 and 0.23, respectively. The primary reason for this phenomenon is that these defects typically exhibit extremely low contrast, and their morphological characteristics closely resemble the complex background texture of solar cell grains, making it difficult for the model to completely separate them from the background during the feature extraction stage. Nevertheless, thanks to the optimization of bounding box regression by the Inner-MDPIoU loss function, the model maintains strong robustness in overall multi-class confusion scenarios, with most defects not being misclassified into other categories (e.g., Broken Gates are rarely mistaken for scratches).

### 5.3. Ablation Study

To rigorously validate the effectiveness of the proposed enhancement modules (C3k2_VSS, SPD-Conv, InnerMPDIoU) within the YOLOv11n object detection framework, we conducted comprehensive ablation studies, with detailed experimental results presented in Table 5. It is particularly noteworthy that the introduction of the loss function InnerMPDIoU does not alter the model’s parameter count or computational load.

After introducing the SPD-Conv module into YOLOv11n, the Precision reached 0.849 and the mAP0.5 reached 0.842, indicating that it contributes positively to detection accuracy, although Recall and mAP0.5:0.95 showed slight decreases. Based on this, the introduction of the C3k2_VSS module led to a significant improvement in Recall, which increased to 0.798, and mAP0.5:0.95, which rose notably to 0.611, thereby enhancing the model’s detection recall capability. Furthermore, when SPD-Conv, C3k2_VSS, and InnerMPDIoU were integrated simultaneously, the model achieved optimal overall performance, with a Precision of 0.872, Recall of 0.819, mAP0.5 of 0.861, and the highest mAP0.5:0.95 of 0.623. Compared with the original YOLOv11n model, these metrics represent improvements of 7.8%, 1.7%, 2.8%, and 5.1%, respectively. This fully validates the effectiveness of the proposed improvements in enhancing detection accuracy, localization precision, and the overall comprehensive performance of the model.

### 5.4. Training Experimental Results of the Improved MambaVSS-YOLOv11n Model

**Figure 11:**
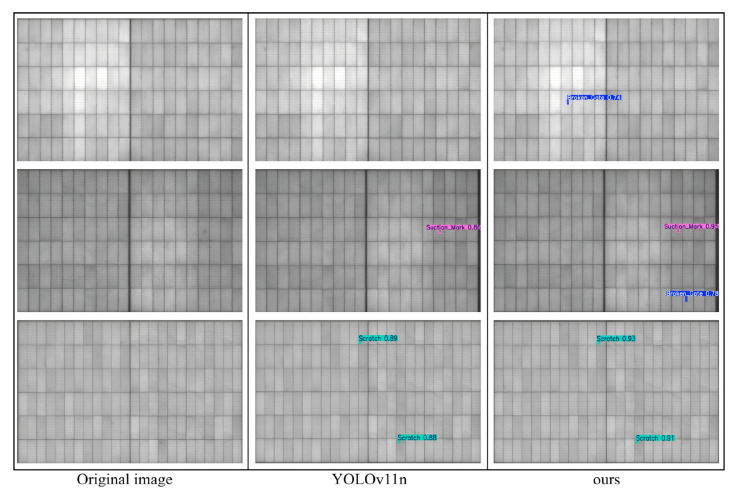
Detection Results Comparison between YOLOv11n and MambaVSS-YOLOv11n.

A comparative analysis of detection results between YOLOv11n and MambaVSS-YOLOv11n is illustrated in Figure 11. To clearly demonstrate the detection performance for subtle defects, we focused on regions prone to missed detection in the comparison figure. The Ours model accurately delineates the defect boundaries thanks to the global perception capability of the VSS module. For low-contrast “scratches” (highlighted in cyan boxes), it exhibits higher confidence and localization precision. This also visually validates the role of the Inner-MDPIoU loss function in improving the accuracy of bounding box regression.

## 6. Conclusions

This study proposes the MambaVSS-YOLOv11n method for multi-defect detection in electroluminescence images of photovoltaic modules. The approach introduces SPD-Conv downsampling to preserve spatial details and integrates the VSS module to reconstruct the C3k2 bottleneck structure, thereby enhancing the model’s ability to capture fine-grained details and model long-range semantic dependencies. By adopting the InnerMDPIoU loss function, the method significantly improves the robustness of bounding box regression for small targets and complex backgrounds while reducing the negative impact of anomalous samples on model convergence. Compared to Transformer-based detectors, this study leverages Mamba’s linear attention mechanism to achieve efficient global modeling under limited data conditions without the need for costly pre-training. Additionally, compared to large CNN models (e.g., YOLOv11-Large) that often sacrifice inference speed for higher accuracy, our model achieves an mAP@0.5:0.95 of 0.637 with only 2.12 M parameters, demonstrating significant engineering advantages in the accuracy-efficiency trade-off.

However, in practical photovoltaic production lines, systems typically require a false negative rate below 0.5% and processing time per image under 30 milliseconds. Our model achieves a recall of 0.824 in the test set and exhibits FPS performance that meets real-time requirements, essentially reaching the application threshold for online detection. Nevertheless, this study has certain limitations: (1) The selective scan operations in the Mamba architecture currently heavily rely on CUDA optimization, and their deployment and inference optimization on edge embedded devices without GPU acceleration (e.g., FPGA or CPU-only industrial computers) require further exploration; (2) The experiments do not include extensive cross-dataset testing, and samples of extremely rare defect categories (e.g., sintering spots) remain scarce, which may affect the model’s generalization capability in extreme anomaly scenarios. Future work will focus on cross-dataset validation, hardware-aware model optimization, and active learning strategies for low-frequency defects.

## Figures and Tables

**Table 1:** Types and Descriptions of Photovoltaic Module Defects.

No.	Defect Type	Defect Description	Defect Quantitative Description
1	Broken Gate	Typically manifests as interrupted or missing areas in electrode grid lines	The break is perpendicular to the finger direction.
2	Cold Solder Joint	Usually appears as irregular areas with locally reduced brightness and blurred edges	The total void area on a single cell exceeds 10% of the cell area.
3	Black Spot	Characterized by black or dark areas at edges/corners, forming sharp contrast with surrounding bright regions	The span of a black spot defect on a single cell exceeds 1/15 of the cell’s diagonal length.
4	Scratch	Presents as linear or irregular low-luminance areas	The length exceeds one-third of the cell length.
5	Microcrack	Appears as continuous faint dark lines	The number of microcracks on a single cell shall not exceed two, and the length of each microcrack shall not exceed one-half of the solar cell side length.
6	Suction Mark	Shows regular circular or oval-shaped low-brightness areas	Nearly circular with uniform gray level internally.

**Table 2:** Experimental environment.

Name	Parameter
Operating System	Ubuntu18.04
CPU	12 vCPU Intel(R) Xecon(R) Platinum 8255C CPU @2.50 GHz
RAM	90 GB
GPU	RTX4090
GPU Memory	24 GB
Programming Language	Python 3.8
Deep Learning Framework	PyTorch 2.0.1 + Cuda 11.8

**Table 3:** Model Training Hyperparameter Configuration.

Name	Parameter
Learning Rate Update	Cosine Annealing
Optimizer	AdamW or SGD-Momentum
Training Epochs	200
Batch Size	16
Early Stopping Patience	50
Pretrained Weights	False

**Table 4:** Performance comparison of object detection models.

Model	Precision	Recall	mAP@0.5	mAP@0.5:0.95	Params	FLOPs (G)	Inference Speed (fps)
YOLOv5n	0.820	0.749	0.831	0.537	2.49	7.8	285
YOLOv6n	0.761	0.487	0.566	0.276	4.15	11.5	210
YOLOv8n	0.723	0.799	0.811	0.529	2.86	8.2	278
YOLOv9t	0.872	0.792	0.828	0.560	1.90	7.6	290
YOLOv10n	0.820	0.677	0.774	0.521	2.61	8.3	275
YOLOv11n	0.794	0.802	0.833	0.572	2.59	6.4	315
**ours**	**0.872**	**0.819**	**0.861**	**0.623**	**2.12**	**8.0**	**288**

**Table 5:** Comparison of ablation study metrics.

No.	SPD-Conv	C3k2_VSS	InnerMPDIoU	Precision	Recall	mAP0.5	mAP0.5:0.95
1	×	×	×	0.794	0.802	0.833	0.572
2	**√**	×	×	0.849	0.791	0.842	0.57
3	**√**	**√**	×	0.859	0.798	0.857	0.611
**4**	**√**	**√**	**√**	**0.872**	**0.819**	**0.861**	**0.623**

## Data Availability

Data will be made available on request.
